# Supplementary material for: Colonization and Biodegradation Potential of Fungal Communities on Immersed Polystyrene vs. Biodegradable Plastics: A Time Series Study in a Marina Environment
Source: J Fungi (Basel). 2024 Jun 18;10(6):428. doi: 10.3390/jof10060428 (PMC11204492; doi:10.3390/jof10060428)
Supplement: Supplementary file 1 [file jof-10-00428-s001.zip › Supplementary_Table_revised_vf.pdf]

Supplementary Table S1. Plastic materials immersed and their intrinsic characteristics

Material	Code	Biodegradability	Transfer of hazardous chemicals	Toxicity on marine organisms*
Expanded Polystyrene (PSE-CP)	EPS-F	No	0.7	0.3
Expanded Polystyrene (PSE-PI)	EPS-I	No	1	0.6
Extruded Polystyrene (PSX-BA)	XPS-O	No	1.2	0.4
Polylactic Acid (PLA)	PLA-F	Yes (industrial composting facilities)	0	0.1
Polylactic Acid + Polybutylene Adipate Terephtalate (PBAT)	PLA/PBAT	Yes (industrial composting facilities)	0	0.1
Poly3-hydroxybutyrate-co-3-hydroxyhexanoate (PHBH)	PHBH-F	Yes	0	0

* The toxicity scores (from 0 to 1) were assessed based on 7 ecotoxicological tests: cytotoxicity of solvent extracts on bacteria and fish cell lines, estrogenic potential (i.e. endrocrine disruption) of solvent extracts, reprotoxicity of solvent extracts on zebrafish early life stages, cytotoxicity of seawater leachates on bacteria, microalgae, and oyster early life stages (Cedre 2022).

Supplementary Table S2. Sequences of ITS, Actine, β-tubuline, 28S primers and their amplification conditions used in this study.

Region/ Gene	Primer name		Sequence (5'-3')	Number of bp	Amplification conditions				
					Cycles	Temperature / Time			
ITS	Forward	ITS4	TCCTCCGCTTATTGATATGC	20		95°C	55°C	72°C	16°C
					1x	5min			
	Reverse	ITS5	GGAAGTAAAAGTCGTAACAAGG	22	29x	1 min	1 min	1 min	
					1x			10min	∞
Actin	Forward	ACT-512F	ATGTGCAAGGCCGGTTTCGC	20		95°C	58°C	72°C	16°C
					1x	8min			
	Reverse	ACT-783R	TACGAGTCCTTCTGGCCCAT	20	29x	15 sec	20 sec	1 min	
					1x			5min	∞
β-tubulin	Forward	Bt2a	GGTAACCAAATCGGTGCTGCTTTC	24		94°C	61°C	72°C	16°C
					1x	5min			
	Reverse	Bt2b	ACCCTCAGTGTAGTGACCCTTGGC	24	35x	1min	1min	1min	
					1x			5min	∞
28S	Forward	NL1	GCATATCAATAAGCGGAGGAAAAG	24		95°C	52°C	72°C	16°C
					1x	2 min			
	Reverse	NL4	GGTCCGTGTTTCAAGACGG	19	36x	1 min	90 sec	2 min	
					1x			*10min*	∞

Supplementary Table S3. Accession numbers related to the most promising isolates.

Isolates	ITS	Actin	β-tubulin	28S
C1589	PP338150	-	PP542031	PP338232
C1591	PP338151	-	PP915709	PP338233
C1666	PP338152	-	-	PP338234
C2218	PP338153	PP542030	-	PP338235
C2281	PP338154	-	PP915710	PP338236
C2559	PP338155	-	PP915711	PP338237

Supplementary Table S4. Alpha diversity index values for each sample for the 18S dataset (natural settings).

Samples	Time	Chao1	Shannon	InvSimpson	Pielou	Number of reads
PLA/PBAT_1_R1	T1	5	0,35	1,17	0,22	129744
PLA/PBAT_1_R2	T2	7	1,65	4,65	0,85	101633
PLA/PBAT_2_R1	T1	3	0,79	1,92	0,72	245716
PLA/PBAT_3_R1	T1	3	0,61	1,57	0,55	161892
PLA/PBAT_3_R2	T2	2	0,60	1,69	0,87	72316
PLA/PBAT_3_R3	T3	4	0,66	1,55	0,48	115505
PHBH_1_R2	T2	4	0,59	1,42	0,42	95063
PHBH_1_R3	T3	1	0,00	1,00	NA	46534
PHBH_2_R1	T1	5	0,99	2,30	0,61	126389
PHBH_2_R2	T2	2	0,12	1,05	0,18	74832
PHBH_2_R3	T3	1	0,00	1,00	NA	197870
PHBH_3_R1	T1	5	1,18	2,94	0,73	107906
PHBH_3_R2	T2	9	1,49	3,39	0,68	35552
PHBH_3_R3	T3	1	0,00	1,00	NA	193154
PLA_1_R1	T1	1	0,00	1,00	NA	31970
PLA_2_R1	T1	10	1,05	2,17	0,46	97801
PLA_2_R2	T2	1	0,00	1,00	NA	1822
PLA_2_R3	T3	2	0,69	2,00	1,00	74210
PLA_3_R1	T1	7	0,85	1,94	0,44	113257
PLA_3_R2	T2	4	0,51	1,30	0,37	105069
PSE-CP_1_R1	T1	6	1,33	3,12	0,74	201168
PSE-CP_1_R2	T2	1	0,00	1,00	NA	16846
PSE-CP_2_R1	T1	7	1,13	2,43	0,58	64029
PSE-CP_2_R2	T2	3	0,83	2,07	0,76	217547
PSE-CP_2_R3	T3	5	1,13	2,48	0,70	118895

PSE-CP_3_R2	T2	2	0,67	1,90	0,96	63689
PSE-PI_1_R2	T2	1	0,00	1,00	NA	168889
PSE-PI_1_R3	T3	2	0,12	1,05	0,17	243384
PSE-PI_2_R3	T3	3	0,69	1,99	0,63	41014
PSE-PI_3_R2	T2	1	0,00	1,00	NA	4240
PSE-PI_3_R3	T3	3	0,63	1,69	0,57	218653
PSX-BA_1_R1	T1	3	0,32	1,17	0,29	37139
PSX-BA_1_R2	T2	2	0,68	1,96	0,98	181218
PSX-BA_1_R3	T3	7	1,60	4,30	0,82	193525
PSX-BA_2_R1	T1	2	0,11	1,05	0,16	89141
PSX-BA_2_R3	T3	6	1,58	4,53	0,88	154124
PSX-BA_3_R3	T3	1	0,00	1,00	NA	222700
Seawater_R2	T2	10	1,75	4,60	0,76	158757

* The quality of the extracted DNA appears to be adequate, with an average 260/280 ratio of 1.9 ± 0.3. Also, our rarefaction curves reach saturation for each sample, clearly indicating that the diversity was comprehensively captured.
