# Supplementary figures and images for: Colonization and Biodegradation Potential of Fungal Communities on Immersed Polystyrene vs. Biodegradable Plastics: A Time Series Study in a Marina Environment
Source: J Fungi (Basel). 2024 Jun 18;10(6):428. doi: 10.3390/jof10060428 (PMC11204492; doi:10.3390/jof10060428)

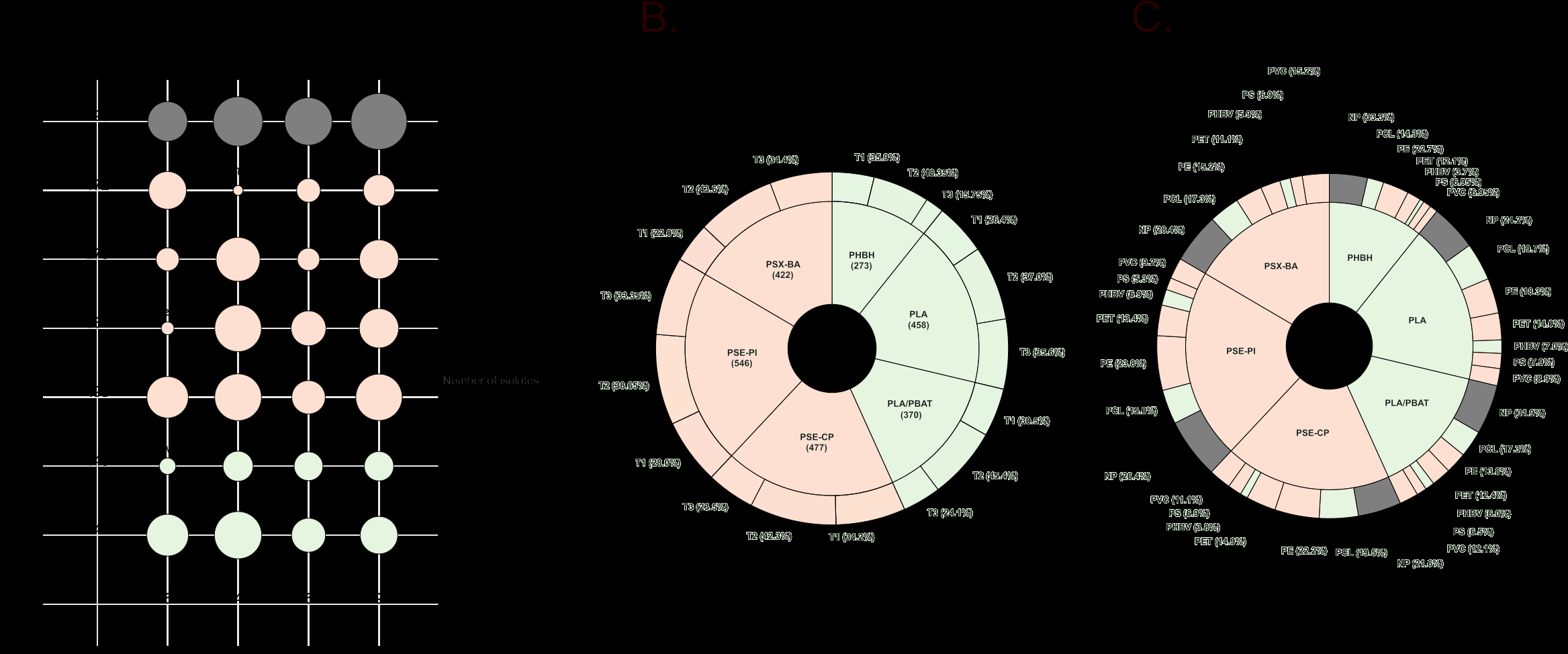

Supplement: Supplementary file 1 [file jof-10-00428-s001.zip › Supplementary Figure S3.tiff]

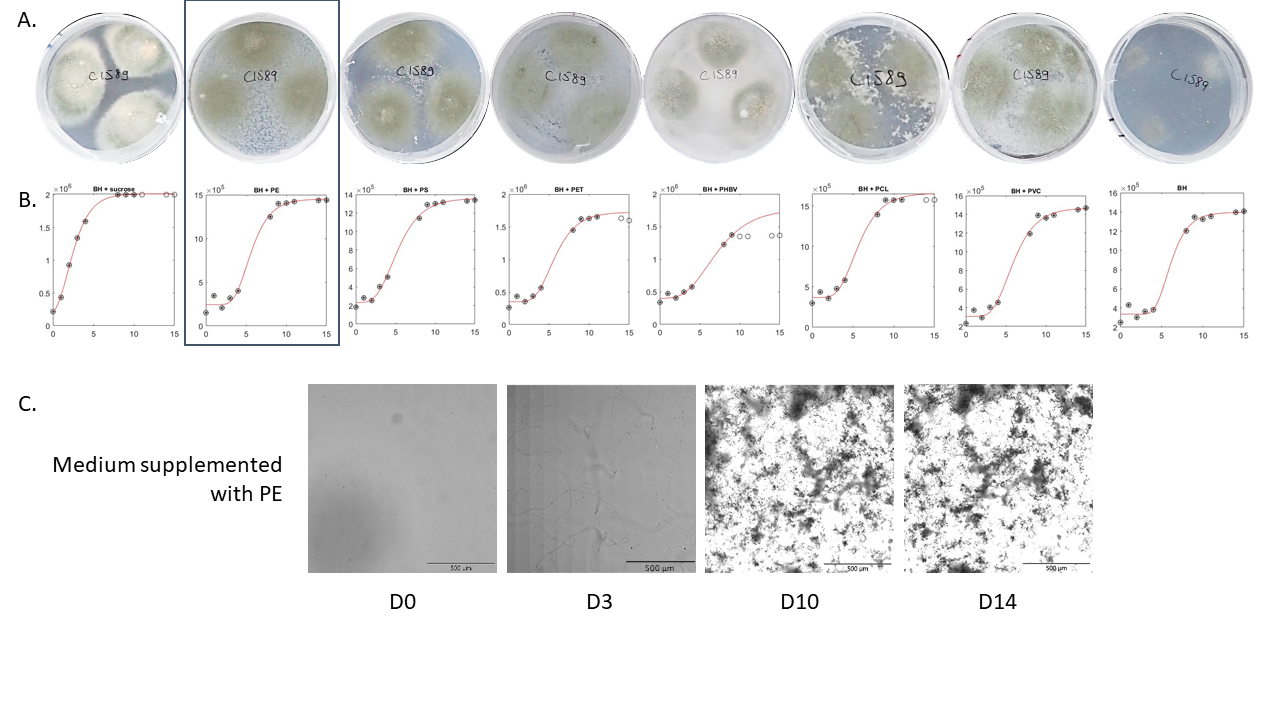

Supplement: Supplementary file 1 [file jof-10-00428-s001.zip › Supplementary Figure S4.tif]

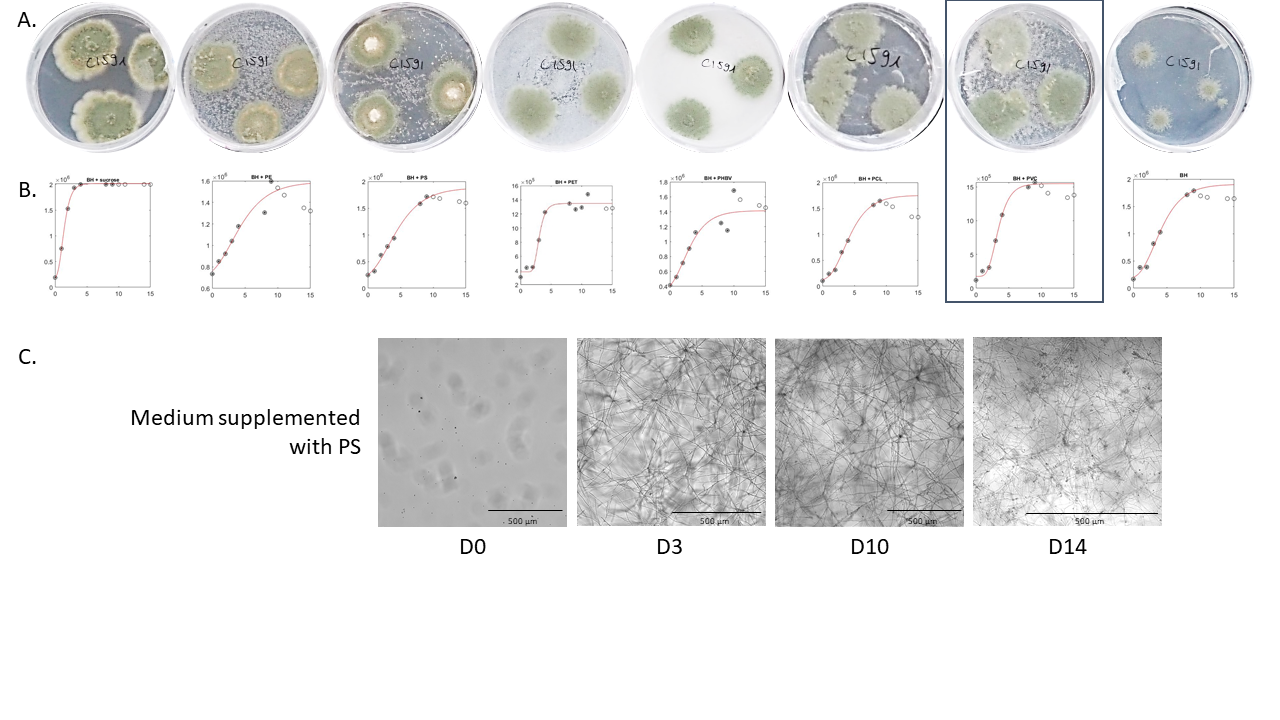

Supplement: Supplementary file 1 [file jof-10-00428-s001.zip › Supplementary Figure S5.tif]

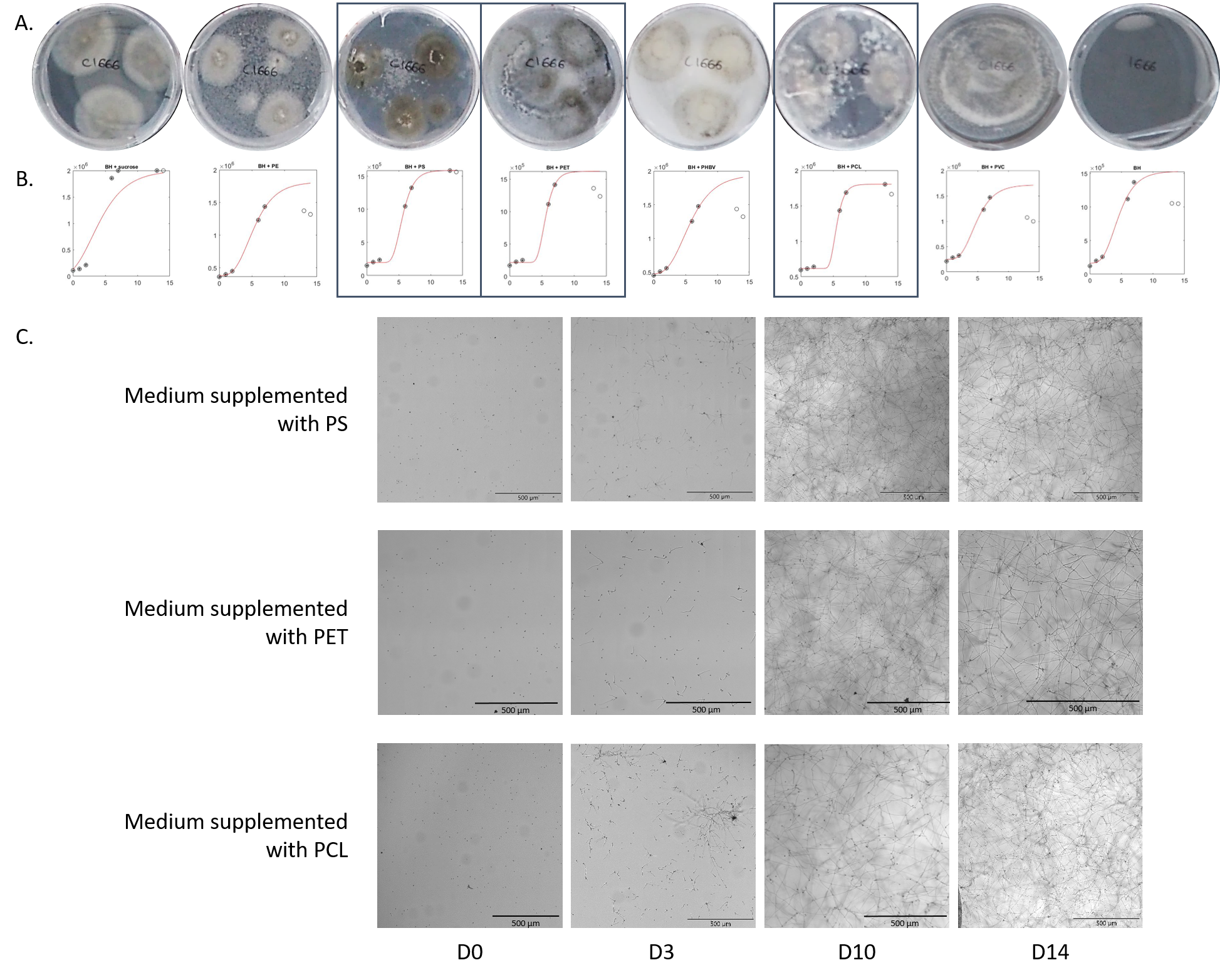

Supplement: Supplementary file 1 [file jof-10-00428-s001.zip › Supplementary Figure S6.tif]

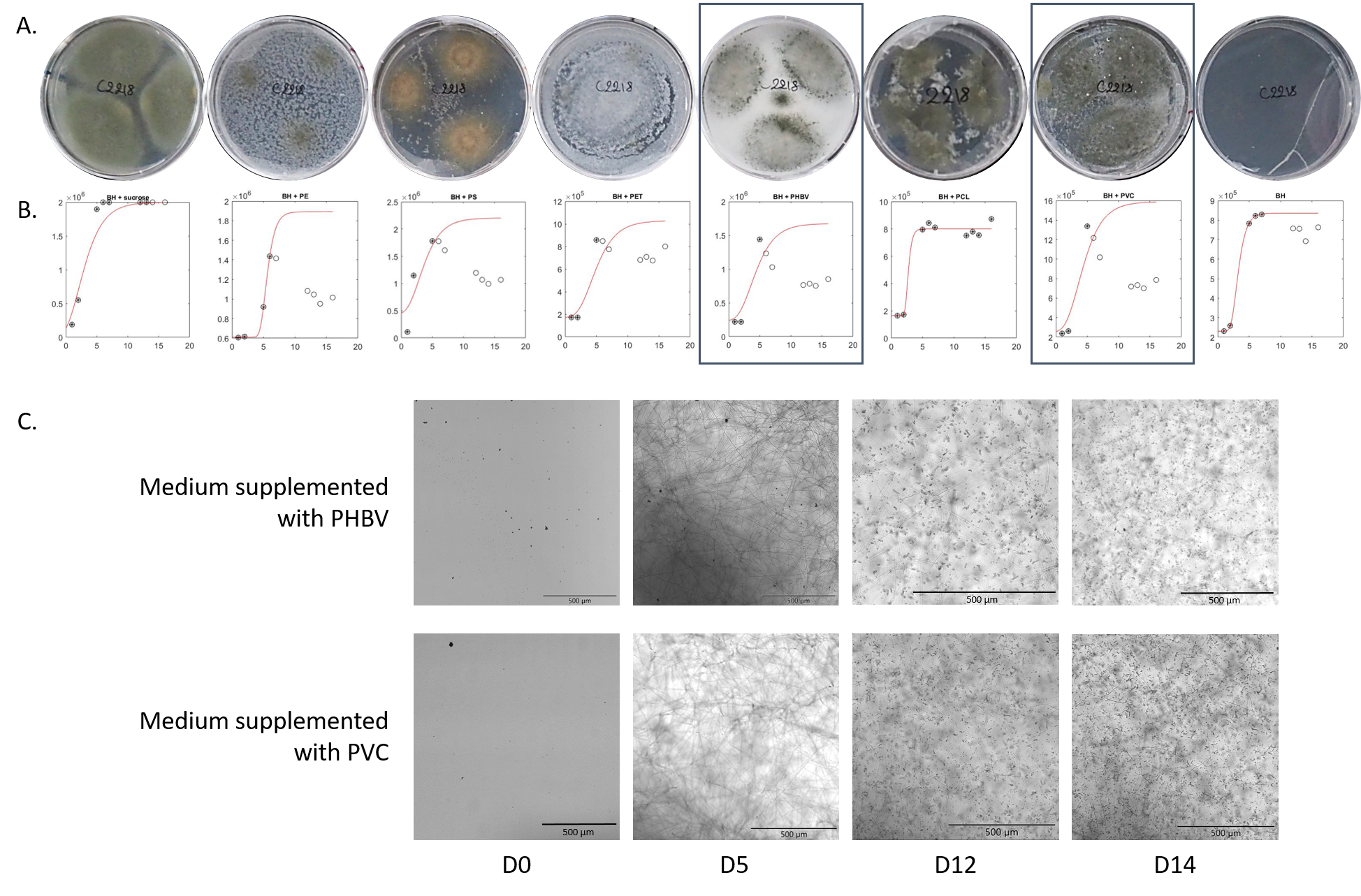

Supplement: Supplementary file 1 [file jof-10-00428-s001.zip › Supplementary Figure S7.tif]

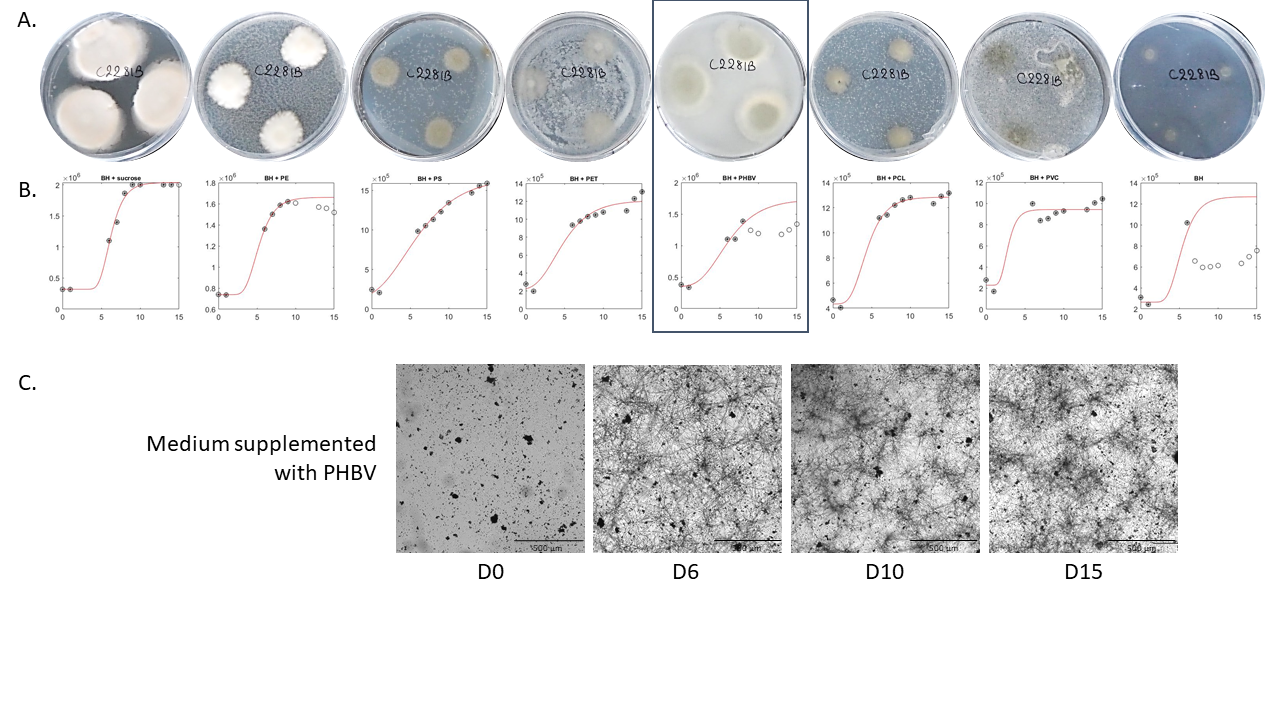

Supplement: Supplementary file 1 [file jof-10-00428-s001.zip › Supplementary Figure S8.tif]

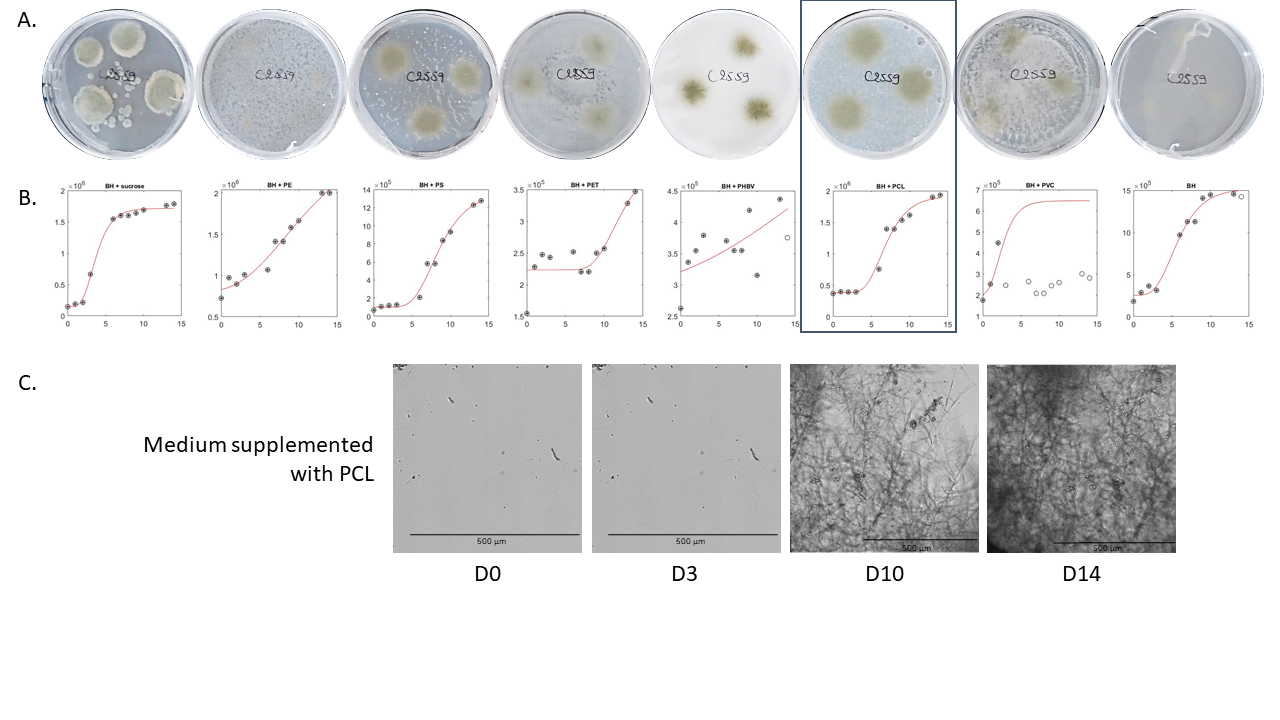

Supplement: Supplementary file 1 [file jof-10-00428-s001.zip › Supplementary Figure S9.tif]
